# How to Think Rationally about World Problems

**DOI:** 10.3390/jintelligence6020025

**Published:** 2018-04-25

**Authors:** Keith E. Stanovich

**Affiliations:** Department of Applied Psychology and Human Development, University of Toronto, 252 Bloor St. West, Toronto, ON M5S 1V6, Canada; keith.stanovich@utoronto.ca

**Keywords:** rationality, intelligence, world problems, meliorism

## Abstract

I agree with the target essay that psychology has something to offer in helping to address societal problems. Intelligence has helped meliorate some social problems throughout history, including the period of time that is covered by the Flynn effect, but I agree with Sternberg that other psychological characteristics may be contributing as well, particularly increases in rationality. I also believe that increasing human rationality could have a variety of positive societal affects at levels somewhat smaller in grain size than the societal problems that Sternberg focuses on. Some of the societal problems that Sternberg lists, however, I do not think would be remedied by increases in rationality, intelligence, or wisdom, because remedy might be the wrong word in the context of these issues. Issues such as how much inequality of income to tolerate, how much pollution to tolerate, and how much we should sacrifice economic growth for potential future changes in global temperature represent issues of clashing values, not the inability to process information, nor the lack of information, nor the failure to show wisdom.

## 1. Introduction

The topic of this symposium is:

If intelligence is truly important to real-world adaptation, and IQs have risen 30+ points in the past century (Flynn effect), then why are there so many unresolved and dramatic problems in the world, and what can psychology do about them?

The topic elicits three reactions from this commentator. I will elaborate on each, but, taken singly, my initial reactions were these:

1. Many world problems have, in fact, lessened over the years of the Flynn effect (world-wide starvation and hunger for example). Perhaps the problems that remain, or that have not lessened significantly, are those that are really difficult to solve, and thus, are most intractable.
2. Intelligence is important, but may not be enough to solve some of the problems. The many real problems that society still faces may be due to deficits of rationality rather than intelligence.
3. Many of the remaining world problems involve complex value conflicts—where people differ in their judgments about the optimal solution. These problems are often framed with a myside bias. On both sides of the political spectrum, many of us see a social issue in which our side has not totally won and define it as a “problem”, when in fact, what we are seeing is not a problem per se, but a social compromise in which neither side has totally won the day.

Most of my commentary will focus on reactions #2 and #3, but for completeness, I will begin with a brief nod to #1.

## 2. Maybe Things Really Aren’t So Bad After All

The framing of Sternberg’s essay sometimes makes it seem as if we are supposed to adopt the default view that the world is getting worse. Although significant problems do remain, there is a good case to be made that we have been increasingly solving social problems throughout the past 100 years and more. In other words, significant progress in solving the world’s problems has occurred.

Steven Pinker’s book *The Better Angels of Our Nature* ([1], see also [2]) shows that a variety of large-scale negative social phenomena have, in fact, been decreasing throughout history—and that the decrease encompasses the period of the Flynn effect (however, see [3]). So, for example, Pinker shows that murder and violence have been decreasing throughout history. Likewise, various types of prejudice have been decreasing throughout history and throughout the period of the Flynn effect. The rights of vulnerable groups, such as children and women, have been increasing throughout history. Domestic violence has decreased, as has crime in general and child abuse in particular. Hate crimes have been decreasing throughout history, as have hunger and poverty. (Pointing this out is not to endorse a cavalier attitude toward the problems that are caused by modernity, an attitude that Gopnik [4] sees in Pinker’s work).

All of these positive developments are the result of cumulative cultural ratcheting [5] enabling change to be biased in a positive direction. These cultural changes may have, in part, resulted from Flynn-like changes in abstract thinking. Indeed, Pinker [1] argues just that. But interestingly, Pinker follows Flynn [6] in arguing that the increase in abstract thinking is the result of the spread of scientific thinking making hypothetical thought more habitual. Such a view is consistent with my argument [7,8] that modernity, in the form of schooling and scientific reasoning has increased decontextualizing thinking styles among the population—and that this increases algorithmic-level functioning by making cognitive decoupling less capacity demanding and unnatural.

From my standpoint, as a researcher studying rational thinking, a causal model that operates in this manner—with rational/scientific culture as a causal influence on abstract reasoning (and hence IQ) becomes especially interesting. Rationality is a cultural achievement. Rational beliefs and actions are supported by strategies and knowledge that were not part of our biological endowment, but were cultural discoveries. The development of probability theory, logic, concepts of empiricism, and scientific inference throughout the centuries have provided humans with conceptual tools to aid in the formation and revision of belief and in their reasoning about action. As a culture, we have been engaging in a progressive cultural critique of the cognitive tools we use to act and think more rationally [7,9]. Although rationality is a cultural product, it has individual effects as well. My research group has long argued that one can measure individual differences in rational thinking [10,11].

This is my preferred place to look for the answers to the problem that Sternberg has set for us in his essay—why, despite the Flynn effect, are there are still so many unresolved and dramatic problems in the world. For Sternberg is certainly right in his target article that there are some big problems facing us and it certainly seems like the 30 extra IQ points that we have gotten from the Flynn effect have not totally solved these problems.

## 3. Rationality and Its Practical Effects

As a cognitive scientist, I am a so-called Meliorist [7,12]—which is someone who believes that there are defects in our thinking and that these defects can be remedied. As a Meliorist, I am concerned about the real-life effects of bad thinking. A Meliorist worries about things: parents who fail to vaccinate their children; the billions of dollars that are wasted on quack medical remedies; the many retirements that are ruined through failures to think through foreseeable financial implications of actions earlier in life; the pyramid sales schemes that sweep through middle-class neighborhoods; the children with reading disabilities who are treated with pseudoscientific methods involving balance beams and tinted lenses when other proven treatments exist; the many people who fail to process the implications of credit card debt; that information about probabilities is misused in legal proceedings (thereby freeing the guilty and convicting the innocent); that clinical psychologists persist in using psychodiagnostic instruments with no proven efficacy; and, that otherwise intelligent prosecutors pursue innocent people because of a theory that was developed too early on the basis of too little evidence.

What we have in many of these examples are cases of smart people acting foolishly—a phenomenon that both Sternberg [13] and I [14,15] have written about previously. People tend to find this phenomenon perplexing, but they really should not. Foolish behavior results when people make poorly considered judgments and take injudicious actions. As I have argued in previous publications, the skills of judgment and decision making are not assessed on IQ tests, so it should not be surprising that a person could have a high measured IQ but have modest or low judgment and decision making skills. There is no paradox in the “smart but acting foolish” phenomenon, because the intelligence construct that the tests actually measure (general mental “brightness”) is not the same as the tendency to make judicious decisions—what most cognitive scientists would call rational thinking. If we were clear about the fact that the two concepts (intelligence and rationality) are different, the sense of paradox or surprise at the “smart but acting foolish” phenomenon would vanish. What perpetuates the surprise is that we tend to think of the two traits as one.

The confusion is fostered because psychology has a measurement device for one (intelligence) but not the other (rationality). Psychology has a long and storied history (of over one hundred years) of measuring the intelligence trait. Although there has been psychological work on rational thinking, this research started much later and was not focused on individual differences. Our research group has tried to remedy this situation by creating a beta version of what a test of rationality would look like—our Comprehensive Assessment of Rational Thinking, the CART [16].

A novice psychology student might be a bit confused at this point—thinking that somewhere along the line, they have heard definitions of intelligence that included rationality. Such a student would be right. Many theoretical definitions of intelligence incorporate the rationality concept by alluding to judgment and decision making in the definition. Other definitions emphasize behavioral adaptiveness, and thus also fold rationality into intelligence. The problem here is that none of these components of rationality—adaptive responding, good judgment, and decision-making—are assessed on commonly used tests of intelligence. In terms of the old psychometric distinction between the measures of typical performance and measures of maximum performance, rational thinking assessments lean more toward the typical performance end of the continuum than do intelligence tests. Many rational thinking task items suggest a compelling intuitive response that happens to be wrong. In these tasks, unlike the case for intelligence tests, the subject must detect the inadequacy of the intuitive response that is automatically triggered. They must then suppress this response while selecting a better alternative. Intelligence tests also tend to present problems that are unambiguously framed by their instructions. Rational thinking tasks, in contrast, often require the subject to choose a particular construal. In fact, it is this design feature that makes the task diagnostic. In a probabilistic reasoning task, the entire point is to see how dominant or nondominant the statistical interpretation is over the narrative interpretation.

The “smart but acting foolish” syndrome that is mentioned above suggests that one of the answers to the topic theme of this symposium (“why are there so many unresolved and dramatic problems in the world, given the Flynn effect”) might be that what society really needs is more rationality in addition to intelligence. I perhaps can embellish this conjecture by expanding upon a thought experiment from one of Baron’s earliest books [17]. Baron (p. 5) asks us to imagine what would happen if we were able to give everyone a harmless drug that increased their algorithmic-level cognitive capacities (discrimination speed, STM capacity, etc.)—in short, that increased their intelligence. Imagine that everyone in North America took a pill before retiring and then woke up the next morning with one more slot in their working memories. Both Baron and I believe that there is little likelihood that much would change the next day in terms of human happiness. It is very unlikely that people would be better able to fulfill their wishes and desires the day after taking the pill. In fact, it is quite likely that people would simply go about their usual business—only more efficiently. If given more short-term memory capacity, people would, I believe: carry on using the same ineffective medical treatments, keep making the same poor financial decisions, keep misjudging risks, and to continue making other suboptimal decisions. The only difference would be that they would be able to do all of these things much more quickly due to their enhanced algorithmic-level computational abilities! I think that Baron is right that, in contrast to the working memory pill, increasing rational thinking skills—processes of accurate belief formation, belief consistency assessment, and behavioral regulation—might really improve our own lives and those of others.

In another book, I have described making the choice between more intelligence or more rationality as asking the question: Would you rather get what you want slowly or get what you don’t want much faster? I agree with some of the thrust of Sternberg’s essay in thinking that society’s focus on intelligence at the expense of rationality has meant that what we have been fostering is the tendency to get what we do not want much faster!

With my “get what you want” phrasing, I am referring here to one of two types of rationality identified by philosophers—instrumental rationality. Most colloquially, instrumental rationality amounts to behaving in the world so that you get exactly what you most want, given the resources (physical and mental) available to you. Somewhat more technically, instrumental rationality can be characterized as the optimization of the individual’s goal fulfillment. Economists and cognitive scientists have refined the notion of optimization of goal fulfillment into the technical notion of expected utility.

The other aspect of rationality that is studied by cognitive scientists is termed epistemic rationality. This aspect of rationality concerns how well beliefs map onto the actual structure of the world. The two types of rationality are related. In order to take actions that fulfill our goals, we need to base those actions on beliefs that are properly calibrated to the world.

When properly defined, virtually no person wishes to eschew epistemic rationality and instrumental rationality. Most people want their beliefs to be in some correspondence with reality, and they also want to act in ways to maximize the achievement of their goals. Manktelow [18] has emphasized the practicality of both types of rationality by noting that they concern two critical things: What is true and what to do. Epistemic rationality is about what is true and instrumental rationality is about what to do. For our beliefs to be rational they must correspond to the way the world is—they must be true. For our actions to be rational, they must be the best means toward our goals—they must be the best things to do. Nothing could be more practical or useful for a person’s life than the thinking processes that help them to find out what is true and what is best to do.

In our comprehensive assessment of rational thinking, the CART [16], we assess aspects of instrumental rationality and irrationality, such as: the ability to display disjunctive reasoning in decision making; the tendency to show inconsistent preferences because of framing effects; the tendency to substitute affect for difficult evaluations; the tendency to over-weight short-term rewards at the expense of long-term well-being; the tendency to have choices affected by vivid stimuli; and, the tendency for decisions to be affected by irrelevant context. Aspects of epistemic rationality that are assessed include: the tendency to show incoherent probability assessments; the tendency toward overconfidence in knowledge judgments; the tendency to ignore base rates; the tendency not to seek falsification of hypotheses; the tendency to try to explain chance events; the tendency to evaluate evidence with a myside bias; and, the tendency to ignore the alternative hypothesis.

Although measures of individual differences in these rational thinking components are correlated with individual differences in intelligence, the relationship is not high enough to warrant the idea that an IQ test provides a measure of rational thinking. The magnitude of the observed correlation leaves plenty of room for dissociations between intelligence and rationality.

One way to illustrate the potential for dissociation is to consider some examples from the literature showing that professionals—all of whom must be well above average in intelligence, have been shown to make dozens of the rational thinking errors that are assessed on the CART. For example, practicing physicians have shown framing effects in real-life medical problems [19]; ignoring the base-rate likelihood has been demonstrated in studies of medical personnel, lawyers, stockbrokers, sportswriters, economists, and meteorologists [20,21]; stock market investors mistakenly think that they can “beat the market” because they fail to appreciate the role of chance [22]; clinical psychologists have been shown to rely too heavily on single-case evidence of low diagnosticity [23,24]; lawyers have been found to be more likely to settle a case out of court when it was described, or framed, in terms of gains rather than equivalent losses [25]; overconfidence has been shown to reduce the earnings of professional traders [26,27,28]; and, physicians and clinical psychologists often fail to engage in diagnostic hypothesis testing [24,29,30,31].

These aspects of rational thinking have been linked to practical real-world behaviors in many different studies. Table 15.1 of Stanovich et al. [16] contains many such examples. The selection of examples mentioned here clearly show that there are many practical ways that the world would be a better place if everyone became more rational. For example, increased rationality would mean that people would deal better with certain risks. More people would wear seatbelts and fewer would text while driving, thus reducing casualties from traffic accidents. Thaler and Sunstein [32] were rightly lauded for showing how environmental changes (nudges) could lead to many similar incremental improvements.

Perhaps some of these improvements might be considered small-bore when compared to the large-scale problems that Sternberg enumerates in the target essay (climate change, poverty, pollution, violence, terrorism, opioid poisoning, income disparities, a divided society). But society seems to appreciate most of these small-bore improvements because many of them are nonzero-sum—a gain in outcome for one participant in an interchange does not entail a loss for another. Perhaps the reason that some of the large-scale problems that Sternberg mentions remain unsolved is because they have more of a zero-sum quality to them.

## 4. Value Conflicts Versus Optimizable Solutions

The speculation at the end of the last section suggests that perhaps we need to form a taxonomy of the different social problems that Sternberg mentions (climate change, poverty, pollution, violence, terrorism, opioid poisoning, income disparities, and a divided society) because they are in different categories. Two problems on Sternberg’s list (poverty, violence) are in the category I discussed in my first section above—things that Pinker [1] shows actually have improved greatly throughout history, including during the period of the Flynn effect (Pinker would include terrorism in this category too, but I will treat it as a more controversial case). Perhaps the improvement was due to some combination of increasing intelligence and increasing rationality (or, additionally, wisdom). These problems have not been reduced to zero of course, but startling improvements have been made in both.

Other issues in Sternberg’s list—such as climate change, pollution, and income disparities—may be of a different type. Perhaps, in some of these cases, what we are looking at are not problems but rather cases of conflicting values in a society with diverse worldviews. Pollution reduction and curbing global warming often require measures that have as a side effect restrained economic growth. The taxes and regulatory restraints necessary to markedly reduce pollution and global warming often fall disproportionately on the poor. For example, raising the cost of operating an automobile through congestion zones, raised parking costs, and increased vehicle and gas taxes restrains the driving of poorer people more than that of the affluent. I have lived in several environmentally progressive cities where the measures invoked to make driving expensive have indeed driven poorer people on to buses, but they have not affected my behavior. I am free to ride mass transit to make an environmental statement, but I am not forced on to it for monetary reasons, like my less affluent fellow citizens.

There is no way to minimize global warming and maximize economic output (and hence, jobs and prosperity) at the same time. People differ on where they put their “parameter settings” for trading off environmental protection versus economic growth. Differing parameter settings on issues such as this are not necessarily due to lack of knowledge [33,34]. They are the result of differing values or differing worldviews.

The point is that such large-scale problems as climate change and pollution control involve tradeoffs, and it is not surprising that the differing values that people hold may result in a societal compromise that pleases neither of the groups on the extremes. But, it is displaying a myside bias to think that if everyone were more intelligent, or more rational, or wiser that they would put the societal setting just where our own setting currently is. There is, in fact, empirical evidence showing that more knowledge or intelligence or reflectiveness does not resolve zero-sum value disagreements such as these [33,35,36,37,38].

The case of income disparities is complex, but it illustrates the same thing—that there is no one point that is the optimal level of income disparity. Disputes about it are value conflicts, and are not conflicts between the intelligent/rational/moral versus the unintelligent/irrational/immoral. Nor are they conflicts between the knowledgeable and unknowledgeable. Certainly it is not hard to look at cross-national statistics on the Gini index and per-capita GDP and conclude that the income inequality in the United States seems to be less than optimal. But, beyond that gross conclusion, there is little that can be said. There is no optimal level of the Gini index for a given country (the Gini index is the most commonly used measure of inequality in economics—where a higher Gini index indicates more inequality).

So, talking about “the problem” of income inequality seems to be somewhat a misnomer when no one knows what the optimal Gini coefficient is. Gini coefficients have been rising throughout the developed world in the last 30 years [39]. We might grant that the coefficient for the United States is too high. But does Australia have an income inequality problem? Does Sweden? How do we know when we have solved this problem? It is unlike poverty, which we want to reduce to zero.

Australia has the same Gini coefficient now that the United States had in 1985. So, however much inequality was a problem in the United States in 1985 (and there was plenty of talk about it being a problem in the Reagan era) then it is to that degree a problem in Australia now. Sweden—which is often lauded for its level of equality—has had the largest increase of all the OECD countries since 1985. Has that country been making massive economic mistakes? Or under some conditions, can a rising Gini index be a good thing? One only has to look at the full set of time changes across all of the OECD countries to see the impossibility of knowing what the optimal Gini coefficient is. If a certain country was exemplary in year X, another country was exemplary in year X + K, because most (but not all) countries have varying indices. Was Denmark the best country in 2010 because it has the lowest coefficient? Are Turkey and Greece the two OECD countries that are most on track because they are the rare countries in which Gini coefficients are decreasing? We might be able to make ordinal statements about income inequality at times—like my stipulation above that it certainly seems like the US index is higher than optimal—but it is not the kind of “problem” that has a maximizable solution, like poverty.

The concept of income inequality involves some particularly tricky trade-offs between values. The present settings of the inequality parameter at any grain size of social organization (world, country, state, county, city) reflect a social compromise resulting from conflicting values, where these values all reflect a particular public good—just like the values of increasing economic prosperity and decreasing global warming both foster public goods, but in a trade-off relationship. Take, for example, the fact that, although income inequality has been increasing in the past couple of decades in most industrialized, first-world countries, worldwide indices of income inequality have been decreasing during the same period [40]. These two trends may well be related—through the effects of trade and immigration [41]. Any such linkage creates value conflicts from the standpoint of, say, a particular citizen in the United States who is concerned about income inequality in their own country. That same person might support aspects of globalization (maximizing free trade and immigration, for example), because they are supporters of decreasing world poverty and world inequality. But, the very same mechanisms that are supporting decreases in world inequality may well be supporting increases in inequality within the United States—which the same person might also deplore. For example, globalization has enabled the shift in manufacturing to poorer countries, which often eliminates the middle layers of the economy of wealthier countries.

A complementary citizen of the United States might maximally prioritize decreasing inequality within the United States, and hence be a supporter of reduced trade and reduced immigration. But such a person might not like the concomitant effect of increasing worldwide inequality. The structure of the world economy might actually prevent maximizing the values of increasing immigration, decreasing income inequality within the United States, and decreasing inequality throughout the world at the same time. These three goals might not all be statistically achievable simultaneously [41,42]. The varying settings of the inequality parameter among countries—and disagreements among people and ideologies—reflect the natural variability of people’s values. Disagreements about the level of the inequality parameter would not disappear if we were to maximize the rationality of the population, or its knowledge—neither would they disappear were we to make everyone wiser.

To take one last example of the paradoxes and complex trade-offs that surround the issue of income inequality (again compared to the much simpler issues of poverty reduction or hunger reduction), consider the following facts about income equality in the United States in the last 30 years: the top 10% of the population in income and wealth has pulled away from the middle of the population more than the middle has detached from the poor [39]. So, when trying to reduce overall inequality—in the manner that would affect an omnibus statistic like the Gini index—we need to make a value judgment about which of these gaps we want to concentrate on more. The obvious answer here for any equality advocate—that we want to work on both gaps—simply will not do. Some of the policies focused on closing one of these gaps may well operate to increase the other gap [43].

When we say that we are against inequality we really have to make a value judgment about which of these kinds of gaps mean more to us. Note that a focus on poverty does not have this trade-off. A focus on poverty tries to raise up the bottom strata of the population regardless of what it does to other levels. Capitalism and industrialization have precisely the effect that I am suggesting here. As countries go from destitute to industrialized, their poverty decreases, but in the earliest stages of this development their income inequality most often increases [40]. Finally, there is the issue of economic mobility—which is different from income inequality. It is possible for mobility to be high even when income equality is low. Indeed, in the United States economic mobility has not changed throughout the period when the Gini index has been rising [44].

In short, some of the social problems that appear in Sternberg’s list do not have a univariate solution. They have multivariate states of stability that are based on citizens of different political persuasions trading off values in different ways (income inequality, climate, pollution). One other problem that Sternberg mentions, terrorism, would seem to be more like one of the simpler cases. One would think that terrorism would be like poverty and violence—a situation where everyone could agree that the optimal setting would be zero. But even in a case like this, there can be value conflicts. Both John Kerry [45] and Barack Obama [46] have argued that we should acclimate to some nonzero level of terrorism in the United States as a part of our accommodation to other values like globalization. Needless to say, not all Americans agree with the Kerry/Obama position that the optimal parameter setting for terrorism is above zero. Thus, even in the case of terrorism, we have value trade-offs that will not disappear once everyone is educated, intelligent, rational, and wise.

In the conclusion section of the target essay, Sternberg adds “a divided society” to the list of large-scale social issues already mentioned (climate change, income disparities, etc.). This, as an issue, is a quintessential illustration of the kind of problem that is going to be most opaque to solution via an appeal to mental faculties, such as intelligence, rationality, knowledge, or wisdom. Political divisiveness in society is by definition due to value conflict. Thinking that political divisiveness can be resolved by increasing any valued cognitive characteristic would seem to be the epitome of myside bias [47]. To put it a bit more colloquially, for a conservative to think that if we were all highly intelligent, highly rational, extremely knowledgeable, and very wise, all divisiveness would disappear because we would then all be Republicans would seem to be the height of myside thinking. Likewise, for a liberal to think that if we were all highly intelligent, highly rational, extremely knowledgeable, and very wise that all divisiveness would disappear because we would all then be Democrats would seem again to epitomize myside thinking [37,48,49,50,51].

A bit of a similar problem crops up when I wrestle with Sternberg’s conceptualization of wisdom. It involves basically two dimensions. One is balancing the common good against one’s own interests where the common good extends to one’s family, one’s community, one’s nation, and the world. The second dimension involves balancing that common good over the long term as well as the short term. Call these two dimensions the social group parameter and the time parameter, respectively. With two different parameters, both on very wide continua, this definition seems to leave plenty of room for value conflicts. Along the time dimension, how long is long and how short is short? Are long and short defined in the same way across all domains? Beyond pure selfishness, the distribution of the social group concern leaves many degrees of freedom. To put it in an oversimplified way, how much less than 100% moral concern to the self do I have to allocate (and where) before I am deemed a wise person? Am I deemed wise if I allocate 50% to myself and 50% to others? And if so, does it matter where I put the 50% allocated to others?

There just seems to be too many degrees of freedom here for this characteristic to be usefully definable, first of all. Second of all, the causal link between a particular allocation of concern for others and how these large-scale problems are supposed to be solved is very unclear. With the social parameter, do I have to use all of the categories of others (family, community, nation, world)? Or is it okay if I put 50% on myself and 50% on my family? And if the latter allocation is deemed unwise, is it worse than putting 80% on myself, 10% on my nation, and 10% on the world? And if that allocation is insufficient, where do I find the godlike judge who tells me what the distribution should be?

Just like the example of the United States and the Gini coefficient, I think that the appeal to wisdom is only going to be helpful in a weak ordinal sense. I mentioned before that I was quite willing to stipulate that the Gini coefficient in the United States seems inordinately high (but then recall that it was very easy to complicate this judgment by appeals to linkages to decreases in the world Gini coefficient). Similarly, studies of temporal discounting in cognitive science would seem to support Sternberg’s assumption that most people are too short-term in their thinking [52,53,54,55]. I can imagine the time parameter doing some work at least in a weak ordinal sense. But the social group parameter is much more problematic. How would we ever know what is a wise allocation of moral concern over self, family, community, nation, and world? This parameter, for example, must be radically contextually dependent. So, what an increase in wisdom would even mean with regard to this parameter I am unsure of, unless again it is just a weak ordinal suggestion that less should be allocated to oneself and more to the other four groups.

I do not wish to present an overly pessimistic view of our present intellectual landscape, however. Work has been done on the possibility of the rational adjudication of conflicting values [56,57,58,59,60]. I myself devoted a chapter of a 2004 book (chapter 8) to the possibility of rationally critiquing goal formation. But, this work has not advanced to a level that we would call prescriptive for the size of the world problems that are the focus of Sternberg’s target article. It is possible, though, that alternative conceptions of wisdom, going beyond those articulated in the target article (see [61,62,63]), might be able to do some prescriptive work. What makes me skeptical that alternative conceptions can actually do that work, however, is the nature of some of the social problems on Sternberg’s list.

At the heart of most concepts of wisdom is an emphasis on balancing values and interests. But, the claim that wisdom can help with an ongoing social issue, like inequality or pollution prevention, is not just the claim that we need more people balancing interests/values. It is the additional (implied) claim that the interests/values on either side of the issue are not already balanced in the optimal way. It is a meta-claim about the proper balance of interests. No conception of wisdom that I know of prescribes exactly where the quantitative balance should be struck regarding an ongoing issue that has already been the subject of much debate (like income inequality).

Wisdom can help bring unrecognized social problems to our attention. But, for problems where people already are consciously aware of the conflicting values and interests, then wisdom is of limited help, unless the conception of wisdom becomes itself a bit like a political position. In such situations, proponents of wisdom as a solution would seem to be presuming that wisdom is more on their side of the issue than the other side. This position (implicitly the position that wisdom is defined as “going more in the direction of my political party on an issue where interests compete”) seems to define wisdom as having a particular social/political content. Defining wisdom as having socio-political content seems to conflict with most definitions of wisdom of which I am aware.

## 5. Conclusions

I agree with the target essay that psychology might have something to offer in helping us to address societal problems. I do believe that intelligence has helped meliorate some social problems throughout history, including the period of time that is covered by the Flynn effect. I agree with Pinker [1] that increases in intelligence may well have helped us deal with some large-scale social problems like violence, poverty, and hunger. I would go further than Pinker though, and agree with Sternberg that other psychological characteristics may be contributing as well. I think that the positive developmental trends for violence and poverty that Pinker attributes to intelligence could be equally attributed to societal increases in rationality during the same period. As I have argued above, rationality is a cultural achievement and its benefits cumulate because of cultural ratcheting. We are more rational than we were 100 years ago, although of course, 100 years ago, we did not have the parallel test of rationality to prove it!

I also think that increasing human rationality could have a variety of positive societal effects at levels somewhat smaller in grain size than the societal problems that Sternberg focuses on. These are areas that are not trivial in their effects on human happiness. People have their lives ruined and families disintegrate because of irrational thinking about financial matters, short-term thinking about their lives, and the innumeracy of various types that we measure on our rational thinking assessment, the CART [16]. For example, risks are evaluated in various suboptimal ways [64] that result in thousands of deaths due to texting and electronics-distracted driving [65]. Irrational thinking sustains pathological gambling and other behavioral problems [66]. Irrational thinking is what has sustained the dangerous anti-vaccine movement [67]. Some of these real-world problems can be remedied by increasing rational thinking. Similarly, financial decisions, medical decisions, legal decisions, and educational decisions might be improved by teaching more people the tools of rational thinking.

Some of the societal problems that Sternberg lists, however, I do not think would be remedied by increases in rationality or intelligence. I do not think that they would be remedied by knowledge or wisdom either, primarily because remedy might be the wrong word in the context of these issues. Issues like how much inequality of income to tolerate, how much pollution to tolerate, and how much we should sacrifice economic growth for potential future changes in global temperature represent issues of clashing values, not the inability to process information, or the lack of information, or the failure to show wisdom. They are fundamental differences in worldview and on such a large-scale basis are not driven by psychological characteristics that can be normatively evaluated.

Of course, by value conflicts I do not mean the absurdly myside ways these are framed in our current debased political culture (“our values differ because the opposing political party is evil and mine is virtuous”). I mean value conflicts in terms of deep philosophical tradeoffs—tradeoffs between things like: equality and liberty; union wages versus health care costs; and, pollution and economic growth. As Isaiah Berlin said upon receiving an honorary degree at the University of Toronto in a ceremony that I attended, “if these ultimate human values by which we live are to be pursued, then compromises, trade-offs, arrangements have to be made if the worst is not to happen. So, much liberty for so much equality, so much individual self-expression for so much security, so much justice for so much compassion. My point is that some values clash: the ends pursued by human beings are all generated by our common nature, but their pursuit has to be to some degree controlled—liberty and the pursuit of happiness, I repeat, may not be fully compatible with each other, nor are liberty, equality, and fraternity” [68].

Nonetheless, there are plenty of problems for psychologists to work on at a smaller grain-size. It is not unimportant to save for retirement, wear a seatbelt, refrain from electronics while driving, consider baserates in decision making, and understand the cost-benefit tradeoffs when taking actions in the world. These are the type of rational thinking outcomes that can improve the world.
